# Meeting of the Fulton County Medical Society

**Published:** 1915-01

**Authors:** R. R. Daly


### FULTON COUNTY MEDICAL SOCIETY.

### Regular Meeting January 21, 1915.

By R. R. Daly, M. D.

Dr. C. C. Aven presented a case of a girl of 17, blind from childhood who began having what seemed to be epileptic attacks in January, 1914. They would last from a few minutes to two or three hours. She did not bite her tongue nor lose control of the sphincters. There was no aura. The history was negative, but Wassermann showed 4 plus. There was slight haemic heart murmur and an infantile uterus which was retroverted. She had never menstruated.

He operated and replaced the parts as far as possible. Then she was placed upon anti-syphilitic treatment. There have been no attacks for over three weeks. Dr. G. D. Ayer said that he operated upon her eyes for congenital cataract and had done a needling followed by linear extraction. She has a low grade of choroiditis, but is able to see fingers at 15 feet. Of course, she has never "fixed" and it will be interesting to note whether she can learn to do this at her age.

Dr. L. M. Gaines said that albumin and casts may occur in any patient who has convulsions and that they are not specially significant.

As to epilepsy, our conception of it has changed recently. It is not a disease entity, but a group of symptoms following a cause to be ascertained in each instance. Among the causes, syphilis is prominent and the Wassermann test is a routine part of the examination in convulsive states. It should be made both upon the blood and spinal fluid. When increased cell count is present in the spinal fluid, it means syphilis of the nervous system. In treatment, these cases should have bromides to soothe them as well as anti-syphilitic remedies.

Dr. G. W. Quillian exhibited specimens from two cases of appendicitis. One had only slight symptoms, but a cell count of 32,600. There was a ruptured appendix and free pus in the abdomen. The other case was chronic in character and came seeking operation. The adhesions were found and eventually the appendix itself was discovered. It was the smallest he ever had seen and measured less than one-half an inch in length.

Dr. L. C. Rouglin read a paper on Hodgkins Disease and exhibited a case.

Dr. C. W. Gould said that he saw the case early in its course and has made two examinations of the blood at Rouglin's request. The boy is certainly improving greatly in appearance under the treatment. The second examination of the blood showed marked decrease in the lymphocyte count.

Dr. Willis Jones said that he saw the boy first in the spring of 1913. He then had but one enlarged gland. It was removed and Dr. Funke said it was tubercular. About three months later he showed other glands and in November the diagnosis of Hodgkins Disease was clear in Jones' mind. The boy was advised to go to Mt. Sinai where treatment had been offered to him.

Dr. J. S. Derr said that he had treated two cases of Hodgkins Disease with the X-Ray. He had used a leather filter, but still had some dermatitis. The glands melted down under the exposures and the children improved.

Dr. M. T. Davis said that recent literature led him to believe that we were all agreed that the disease was diphtheritic in origin and that he was surprised to note a divergence from that opinion now. He believes that an autogenous vaccine would be of avail in treatment as well as the use of benzol.

Dr. F. G. Hodgson said that extensive treatment with radium had been successful.

Dr. H. I. Battey said that in Billing's Clinic in Chicago, benzol was used in large doses in this disease with success.

Dr. J. E. Paulin said that the case had come into his hands from Dr. Nicolson showing that it had been going the rounds. At that time, there were several glands enlarged, but they appeared to be tubercular. The differential diagnosis is not easy. The boy remained under his observation but a short time. Arsenic and the X-Ray seem to make the best treatment.

Dr. R. T. Dorsey said that leucocytosis is not significant of Hodgkins Disease. The increased lymphocyte count Rouglin reported appeared to him to be more significant of the disease in its acute stage.

Dr. M. T. Pruitt said that the boy visited his clinic in the Anti-Tuberculosis Association and that he then had not only several glands enlarged, but he ran a temperature for several weeks. The glands grew smaller in size under tuberculin.

Dr. Funke said that he would look at his former slides again and he hoped that if there were ever another operation upon the boy, he would have a chance to make further study of the glands. There is no typical blood picture of Hodgkins Disease.

Dr. Rouglin said in closing that two months before the improvement had begun, there could have been no doubt of the diagnosis. Chronic Hodgkins Disease often runs temperature. The blood picture had been modified by the various treatments the boy has had.

He has letters from several investigators saying that the pathology is undetermined and that it is by no means settled that diphtheria or allied bacillus has anything to do with the trouble.

Dr. T. H. Hancock read a paper on Three Surgical Myths —Spinal Concussion, Displaced Uteri and Sacro-Illiac Joint.

Dr. Willis Jones said that he has had considerable experience with "Railroad Spine," and that there is almost constant perpetration of fraud at the court houses in this matter. Recently a man was injured and given $30,000 damages. At a new trial he said he had lost bladder control and sexual function. He was followed when he made a visit to a nearby city and was discovered in sexual relation with a prostitute there. Many people receive slight injuries which seem to be followed by conditions that must have been there prior to the accident. They mistake the cause and bring suit in fairly good faith. Others are manifestly fraudulent. He told of a lady who was struck by a small piece of plaster falling from a ceiling in a store. It was brushed from her shoulders and nothing was thought of it at the time. The next week, however, she had acute salpingitis and peritonitis and spent many weeks in the hospital. She claimed to be paralyzed in the lower extremities, but reacted strongly when a pin was unexpectedly thrust into her foot. The case was settled for $10,000 rather than go to court and continue a long fight. The lady received $1,600 after the lawyers were paid. The next day she walked to a cab, went to the railway station and boarded a train unaided.

Dr. J. S. Derr said that Goldthwaite showed that Sacro-Illiac luxation was a surgical entity and demonstrated it by the X-Ray. Moreover, Dr. Hoke used manipulation to reduce the luxation. He protests against the idea that it is always a myth.

Dr. J. E. Sommerfield said that in his own case, it was no myth. He had been operated upon several times, for the reduction of his dislocation and still has distress in that region. The legal proceedings have had no influence, beneficent or otherwise, upon him.

Dr. F. G. Hodgson said that he had cases of permanent luxation in this region without any suits connected with them.

Dr. J. L. Campbell described a case of abscess between the sacrum and illium with loose bone at the posterior superior spine. The sinus extended along the cocyx. It was X-Rayed by Derr and was seen by others. The pus was evacuated and the finger could be passed between the bones where the joint had been. The man is still on crutches.

Dr. L. C. Fischer said that he had seen this same case and he could not find a dislocation. The man had caught a street car and had swung on to the step. Five days later he had pain and subsequently disease, but Fischer cannot believe it was due to this injury.

Dr. W. S. Goldsmith said that his experience has been like Hancock's on the corporation side. As a rule, sacro-illiac cases are frauds and imposters. When cases are reported as injured and pictures are taken, they do not show dislocation. In cases where there are real dislocations the patients do not have to sue for the corporations prefer to pay honest damages. He has noted that a man hurt in his own yard, cranking his own machine or doing something of the kind, gets well. There is no chance for the damage suit. Juries will take the word of seemingly injured men as against the corporation's, as a rule in his experience.

Dr. L. C. Rouglin said that the psychologic factor in these cases is not always a fraud, though it may lead to error. His own father was hurt by a train and then heard what some doctors who examined him said. He followed the indirect suggestion and was pretty bad off until his son told him he would get well and that he was in good shape. He recovered.

Dr. Hancock closed and said he was discussing only permanent sprains. Sprains will get well if they are real and properly cared for. He said that Campbell's case was on crutches for a sprained ankle prior to the injury complained of and was helped on to the car by the conductor when he attempted to board it as it started. Another case stepped on an elevator a few inches down from the floor. He walked a mile and then went to bed and made a claim for permanent injury. A lady fell in the street and claimed misplaced uterus. She has pus-tubes.

Dr. R. T. Dorsey read a paper, "A Preliminary Report on Pellagra—A Theory as to Its Origin, A Suggestion as to Its Therapy."

Dr. E. L. Griffin said that he had many cases of Pellagra that ate anything and everything and that he could not believe that a restricted diet could be the cause. He recently operated on a man who had no gall bladder. The abdomen was full of bile and he had Pellagra.

Dr. G. C. Mizell said Dorsey's paper was full of facts, but he does not agree with the conclusions. Pellagra has greatly increased in the last decade or two and prior to that time we certainly had many one-sided diets. Unbalanced diet must be ruled out. It is as Goldberger says either some particular thing we are eating or something we are not eating that causes the disease. It is not just mere lack of balance. Mizell believes and thinks he can prove that the disease is due to cotton seed products used as food. The source of lipoids is not clear and their chemistry is complex and multiform. Fats are present everywhere in the nervous system and may act as insulating material for the nerve fibres. When these break down, the nerve currents get crossed and confusion of function results or there is irritation of the nerve by the morbid material. This irritation is central or peripheral or both, depending upon the amount of the destruction and the character of the metabolism. It will account for all the symptoms.

Dr. Willis Jones said that as a rule surgeons will find Pellagra developing in many cases after ether anaesthesia. This would support the theory of the essayist.

Dr. E. C. Thrash said that we should be careful in not jumping at conclusions in this matter. It has been shown that nerve cells themselves have been destroyed. It is a toxic disturbance, but its source has not been proved. The central cells are those most affected. He does not believe that the effects are due to destruction of the nerve sheaths.

Dr. E. C. Cartledge said that it had been his observation that Pellagra had started after operation or some other unusual condition and that it must have been latent for some time before this.

Dr. M. T. Davis pointed out that Goldberger had showed the need of food with high proteid contents such as beans and peas.

Dr. Dorsey closed by explaining his plan for further investigation of the subject along the lines of his essay and promising to give the results as soon as they were available in the spring.

---

It is far better to introduce a non-metallic catheter into the bladder with forceps than with the fingers, however carefully one may "scrub up."—American Journal of Surgery.

---

Speed is of great importance in thyroidectomy for exophthalmis goiter, but also important is deliberation in so placing the ligature around the inferior thyroid vessels that the inferior laryngeal nerve is not included.—American Journal of Surgery.

---

A convenient means of protecting the hand from soiling a rectal examination consists in passing the index finger through a small opening in a square of gauze and applying a rubber cot pulled down over the gauze at the base of the finger.—American Journal of Surgery.

